# The Health Effect of the Number of Children on Chinese Elders: An Analysis Based on Hukou Category

**DOI:** 10.3389/fpubh.2021.700024

**Published:** 2021-11-16

**Authors:** Cuihong Long, Jiajun Han, Chengzhi Yi

**Affiliations:** ^1^School of Economics, East China Normal University, Shanghai, China; ^2^School of International and Public Affairs, Shanghai Jiaotong University, Shanghai, China

**Keywords:** lower fertility, hukou system, elderly health, number of children, China

## Abstract

Based on the 2018 China Health and Retirement Longitudinal Study (CHARLS 2018), from the perspective of urban-rural disparity, this paper investigates how fertility affects Chinese elders' health. We exploit the enactment of the one-child policy in 1979 to construct instrumental variables capturing the health effect of having only one child rather than multiple children. The empirical results show that the health condition of rural elders having only one child is worse than elders having multiple children, while the negative health effect of lower fertility becomes statistically insignificant for urban elderly parents. After considering the selection on both levels and gains, the results are still robust in marginal treatment effect (MTE) estimation. We investigate the potential mechanism in four ways, the results suggest that having only one child instead of multiple children depresses the upstream intergenerational transfer payments more for rural parents; ameliorates offspring's educational attainment more for urban parents; improves housing conditions more for urban elders; and decreases the visit frequency of children to both urban and rural parents. Our findings have important implications, in the context of increasing population aging, the urban-rural inequality caused by the hukou system has been magnified by the declining fertility rate. The Chinese government should pay more attention to rural elders with only one child, and more public-funded socioeconomic resources are needed for one-child parents in rural areas to improve their health. Moreover, the empirical results also imply that urbanization in China may be able to soften the health deterrent effect of lower fertility.

## Introduction

China is the most populous developing country in the world. With the population rapidly aging across China, many scholars have been attracted by the potential elements affecting elders' health (1, 2). Some researchers are concerned about the impact of individual micro-factors such as income, education level, and Internet use on the health of the elderly (3, 4); several scholars pay attention to the health effect of meso-level factors such as social capital, social support, and social network on the elderly (5, 6); there are also some studies concentrating on the health effect of macro policy factors such as the household registration system (also known as hukou system), social security, and public services on the elderly (7, 8). In general, the existing studies, whether from micro, meso, or macro level analysis, all provided important insights for advancing the understanding of elderly health and its influencing factors.

The research regarding the health effect of the number of children on elders' health is relatively insufficient. Since the late 1970s, China has implemented a strict one-child policy on the Han majority for a long time, and the number of one-child families has increased incessantly. Nowadays, more and more one-child parents have entered old age, and their health status deserves more attention. The hukou system has colonized its role of allocating various socioeconomic resources across mainland China since the 1950s, and the urban-rural dualization development mode has been forged accordingly, putting agricultural hukou holders at a disadvantage in accessing resources such as education, medical services, housing, social security, etc. (9, 10). In the context of the urban-rural dualization, the social and medical resources that rural hukou holders can access are far more scarce than their urban counterparts, consequently, children are more irreplaceable for rural parents seeking old-age support. Meanwhile, with the advancement of urbanization, many rural young adults migrate to cities in order to seek economic opportunities. However, due to the restrictions the hukou system has exerted, rural elders cannot migrate to cities with their children, and therefore the number of empty-nest elders living in rural areas has continuously risen. Under the dual insufficiency of social resources and children, rural elders with only one child may face higher risks in various aspects of old-age support, their health condition deserves more extensive attention from all social circles. So, how does the number of children affect the health of the elderly? Through what channels does it work? The answers to the above questions could lead to better understanding of the health effect of fertility on elders, and shed new light on facilitating a healthy aging process.

Under the background of urban-rural dualization, from the perspective of different hukou categories, and drawing support from the latest released CHARLS 2018, we provide empirical answers to the above questions by exploiting the implementation of the one-child policy in 1979 to construct instrumental variables (IVs), and further employ two-stages least square (2SLS) and marginal treatment effect (MTE) techniques.

## Literature Review

### The Urban-Rural Elderly Health Inequality Led by the Hukou System

Before the reform and opening-up of China, the “scissors gap” system defined the developmental pattern throughout mainland China, and the authority depressed the price of agricultural goods boost the relative price of industrial goods, accumulate industrial capital, and further facilitate rapid industrialization. Although the wages of urban workers in industrial sectors had also been trimmed, the urban government provided them with certain social welfare, such as education of children, housing allowance, and medical services, which were far beyond rural residents' accessibility. Consequently, the real income of urban citizens was significantly higher than their rural counterparts, which created a strong motivation for migrating to cities among rural residents. In order to preclude the massive internal migration and the potential collapse of the “scissors gap” developmental pattern, a household registration system (also known as the hukou system) based on birthplace and lineage emerged as the authority required. With the reform of this system, as market-oriented economy gradually renewed the mainland, the restrictions the hukou system imposed on internal mobility gradually lightened. However, the hukou system still dominates social resources allocation to a certain extent, and in large cities where the population migrates to, some important social welfare and public resources are still only available for citizens with local hukou, and those with agricultural hukou could be denied in accessing local government-funded welfare (10). Existing literature has substantiated that the multi-dimensional social welfare disparities caused by the hukou system have adversely affected the health of agricultural hukou holders (11, 12). Intertwined with these urban-rural disparities, the intensified aging process has made the health status of agricultural elders worse than their non-agricultural counterparts (8, 13).

Song and Smith (12) summarize four principles explaining the health effect of the hukou system: (1) Historical time and place, huge differences exist in the growth trajectory between urban and rural hukou holders, rural hukou holders always lack medical and educational resources during their growth stage, in both quantity and quality, which contributes to worse childhood health compared to urban hukou holders. For instance, Hu (14) found that completing junior high school would improve urban hukou holders' health efficiently, in contrast, only when rural hukou holders completed senior high school could the improvement effect of education on their health be observed. This is because schooling in rural areas is always inferior to that in urban areas. (2) Timing of lives, rural hukou holders tend to access health care and value personal hygiene at a later life stage, adding to lower health than urban hukou holders. Chen et al. (15) point out that China's social old-age medical security system is far from flawless, the problem of “valuing the city over the countryside” is particularly prominent, therefore, the probability of rural elders enjoying medical insurance is much lower than that of urban elders. (3) Linked lives, adult children are the primary old-age support for rural elders, as more and more rural young adults migrate to cities in search for better economic opportunities, the left-behind rural elders tend to show lower health status and more severe depressive symptoms. In addition, Wang et al. (16) found that the hukou category is the critical indicator for the choice of old-age care. The rural elders mainly rely upon their children to provide old-age support, while the urban elders mostly depend on their own pensions. (4) Human agency principle, urban hukou holders always possess more work opportunities and higher socioeconomic status, according to cumulative dis/advantage theory, various inequalities created by the hukou system could accumulate over the life course, eventually leading to a significant health gap between rural and urban hukou holders in the middle and late life stage. Through natural experimentation in Beijing, Afridi et al. (10) found that making students' hukou status salient evokes the inferior background of rural migrant students, impairs their confidence, and further significantly reduces their performance in the assigned task compared to their peers with local urban hukou. The accumulation of dis/advantages from childhood will inevitably affect the health level of adulthood. Chan and Buckingham (17) reveal that a series of reforms belie the significance of the hukou system, which remains intact and potent in determining people's access to various socioeconomic resources, and entrenching the urban-rural divide.

Ge et al. (18) indicate that the aging process in China has far outpaced not only most developing countries, but also many developed countries. Successful aging must be healthy aging, which is no longer just a future-oriented policy idea, but a pressing strategic option. In recent years, with the deepening of China's aging, sociologists and economists have extensively discussed the factors affecting elders' health. On the macro level, the health disparity driven by the hukou system have been widely documented (8, 12, 13). The inequality formed on the macro level spontaneously impacts the health effect of micro-level factors. For example, Li and Zhao (19) found that the improvement effect of education on the health of urban hukou holders is significantly greater than that of rural hukou holders. Zhao and Liu (20) demonstrate that Internet usage could ameliorate urban elders' health; this health-improving effect is insignificant for rural elders.

Thus, it can be seen that rural elders might be exposed to higher health risks relative to non-agricultural elders. The tighter socioeconomic resource constraints facing rural elders may make their health more sensitive to the decline in the number of children.

### One-Child Policy and the Health Effect of Depressed Fertility

After the termination of the Cultural Revolution^1^ in 1976, China's authority was shocked by the fact that although the Chinese population increased by almost 50% from 1955, the grain production in 1977 still stagnated at its 1955 level. So, China's authority enacted the one-child policy to rigorously limit the children Han majority families could have (21). As the birth-controlling policy gradually strengthened its grip throughout mainland China, the number of one-child families grew continuously, the burden of population has eased as a result. However, as one-child parents gradually entered into their old-age stage, a series of social problems caused by low fertility have stirred concerns among many sociologists. Chen (22) points out that a one-child family holds natural structural deficiencies and systemic risk. In developing countries where social security and public welfare are relatively insufficient, the sudden reduction in the number of children is likely to reduce the necessary support for the elderly, thereby impairing the health of the elderly. Guo (1) found that parents with multiple children have higher life satisfaction and lower depression compared with parents with only one child. Xv and Feng (23) suggest that China's one-child families face many risks, the deficiencies in economic and emotional support are especially prominent. Liu et al. (2) reveal that only-child parents in rural areas have lower incomes than parents with multiple children, and showing worse physical and mental health. Ma (24) thinks that an important pathway for children to affect their parents' health and cognitive abilities lies in the financial support provided by children to their parents. Oliveira (25) shows that the amount of upstream intergenerational transfer payments was associated positively with the number of children. In other words, parents with only one child receive significantly less intergenerational transfer payments than parents with multiple children. Wang et al. (16) point out that compared with the urban elderly, the rural elderly are more inclined to choose their children as the basis for providing future elderly care. Sun (26) reveals that the average income of the non-agricultural elderly is 4.5 times that of the agricultural elderly. Judging from existing literature, the negative effect of low fertility on upstream intergenerational transfer payments may create a health gap between parents having only one child and parents having multiple children.

Furthermore, the change driven by lower fertility may not be all bad for parents, for instance, the “quantity-quality tradeoff” theory has been verified by many scholars against China's context; raising fewer children would translate into higher educational attainment for the offspring within the family (27, 28), and children's better educational achievement would improve parental health conditions (24). Torssander (29) reveals the significantly negative association between the education of children and parental mortality risk in Sweden. In addition, scholars in rich countries highlight the motherhood penalty in the labor market, in which fertility often curtails mothers' competitiveness in her career (30, 31). Ruppanner (32) finds that births pose more time pressure on mothers than fathers in Australia, which may explain why mothers tend to concentrate on part-time work. Besides, lower fertility also means loosened pecuniary budget constraints facing a family, parents thus have a chance to improve their life quality and curb the negative impact of decreased fertility. However, dwindling intergenerational contact frequency may result from having fewer children, Chen and Fang (33) find that birth controlling did reduce contacts and visits from children. Therefore, decreased fertility may have a mixed effect on parental health; the hukou system may even further complicate the results.

Through reviewing existing literature, we find that there are few studies that explore the impact of the number of children on parental health from the perspective of the hukou system. From this, we draw support from the latest released CHARLS 2018, and explore the above question by exploiting the enactment of the one-child policy in 1979 to construct IVs. We have contributions in the following aspects: (1) Considering the urban-rural dualization in China, we investigate the health effect of the number of children on urban and rural parents, respectively, which supplements previous research. (2) We exploit the one-child policy enactment in 1979 to construct IVs, the empirical strategy we employed shares some characteristics of a natural experiment, which effectively assuages endogenous concern. (3) In addition to the well-known IVs method, we also employ the MTE approach, solving problems of selection on both levels and gains, which makes our results more robust.

## Data

We capitalize upon the 2018 wave of the China Health and Retirement Longitudinal Study (CHARLS 2018) to complete our research. CHARLS mainly collects national representative samples of Chinese citizens aged 45 and older to facilitate scientific research related to elderly people, which is supported by Peking University, the National Natural Science Foundation of China, the National Institute on Aging, and the World Bank. The baseline of CHARLS was introduced in 2011 and incorporates ~10,000 households and 17,500 individuals across 150 counties/districts and 450 villages/resident committees. CHARLS is suitable for our research, it contains copious indicators pertaining to individuals' demographic characteristics, family information, and health status, etc. As aforementioned, the one-child policy mostly targeted the Han majority, and ethnic minorities still had the rights to have two children. Moreover, life expectancy for Chinese men is 73.64, and the average for Chinese women is 79.43, incorporating individuals over 80 years old may cause survival selection bias (34), and previous research studying Chinese elders' health focuses on people aged 80 and below (35), so we limited our sample to those who were between 50 and 80, belonged to the Han majority, and had at least one child in CHARLS 2018. Table 1 presents the descriptive statistics of variables satisfying our sample restriction standards in this research.

**Table 1:** Descriptive statistics of mainly used variables.

**VarName**		**Obs_urban**	**Mean_urban**	**Obs_rural**	**Mean_rural**	**Difference**
adl_index	Activities of daily living index, composed of seven specific indicators (Cronbach's α = 0.7932; overall Kaiser-Meyer-Olkin (KMO) value = 0.8712), with a higher value indicating better health condition	3,116	86.917	11,827	82.693	4.224^***^
iadl_index	Instrumental activities of daily living index, composed of six specific indicators (Cronbach's α = 0.8149; overall Kaiser-Meyer-Olkin (KMO) value = 0.8444), with a higher value indicating better health condition	3,116	94.955	11,833	92.495	2.460^***^
only_child	Having only one child = 1, having multiple children = 0	1,874	0.340	6,805	0.099	0.241^***^
I[c_i_ ≥ c_0_]	The birth year of the first child, after or in 1979 = 1, before 1979 = 0. I[.] is an indicator function	3,137	0.735	11,874	0.742	−0.006
I[c_i_ ≥ c_0_] × (c_i_-c_0_)	The interaction item combining whether the first child born in and after 1979 with the distance between the birth year of first child and 1979, i.e., I[the first child born in and after 1979 ≥ 1] × (the birth year of first child−1979)	1,871	3.897	6,800	3.614	0.283^*^
Agrihk	Hukou category, agricultural hukou = 1, non-agricultural hukou = 0	3,137	0.000	11,874	1.000	−1.000
Female	Gender, female = 1, male = 0	3,137	0.479	11,874	0.529	−0.050^***^
Age	Respondents' age in 2018	3,137	63.392	11,874	62.259	1.134^***^
Education	Degree of education, illiterate = 0, completed elementary school and below = 1, completed middle school = 2, completed high school and above = 3	3,137	1.916	11,874	1.087	0.829^***^
Single	Marital status, single = 1, having a partner = 0	3,137	0.130	11,874	0.128	0.002
CPC	Political partisanship, the Communist Party of China member = 1, others = 0	3,137	0.223	11,874	0.063	0.160^***^
Sibling	Number of siblings	1,713	3.917	6,495	4.092	−0.175^***^
Insurance	Which type of health insurance could you access and benefit from it? No insurance = 0, new rural cooperative medical insurance = 1, urban and rural resident medical insurance or urban resident medical insurance = 2	3,128	1.071	11,852	0.629	0.442^***^
Pension	Which type of pension do you currently receive, expect to receive, or contribute to? Haven't participated in any pension scheme = 0, new rural resident pension = 1, urban and rural resident pension or urban resident pension = 2, basic pension for enterprise employees = 3, public pension for public servants or institution employees = 4	3,073	2.565	11,281	1.133	1.433^***^
Alcohol	Did you drink any alcoholic beverages in the past year? How often? None = 0, less than once a month = 1, more than once a month = 2	3,117	0.687	11,837	0.588	0.100^***^
Smoke	Have you ever chewed tobacco, smoked a pipe, smoked self-rolled cigarettes, or smoked, cigarettes/cigars? Yes = 1, no = 0	3,117	0.444	11,841	0.435	0.009
lntrans_from	The natural logarithm of the amount of intergenerational contact received from offspring during the last year	2,996	0.835	10,981	0.263	0.572^***^
Housing	The housing condition factor, covering seven specific indicators (Cronbach's α = 0.6383; overall KMO = 0.7626), with a higher value indicating better housing condition	1,582	6.409	6,302	6.756	−0.348^***^
mean_edu	Average number of years of education for children	3,110	0.542	11,821	−0.135	0.676^***^
mean_vispermon	Average number of days children not living with you/visit you in person per month	1,641	12.096	6,492	8.750	3.346^***^
Son	How many sons who are still alive do you have?	1,576	7.267	6,272	4.842	2.425^***^
Daughter	How many daughters who are still alive do you have?	3,137	0.715	11,874	0.901	−0.186^***^

*** *p < 0.001*,

* *p < 0.05*.

We select seven specific indicators in CHARLS 2018, using the principal factor method of iterative common factor variance to construct the factor of activities of daily living (ADLs) (Cronbach's α = 0.7932, overall KMO = 0.8712). According to Hamilton (36), the factor analysis using iterated communalities could find the latent dimensions that best explained correlated patterns among variables. The same method is used again to construct factor measuring people's housing condition to examine the potential mechanism. After factor-rotated, we adopt range standardization to convert this factor to a continuous index ranging from 0 to 100, with a higher value indicating a better health condition (37). The seven specific questions are: Do you have any difficulty with (1) running or jogging about 1 km; (2) getting up from a chair after sitting for a long period; (3) climbing several flights of stairs without resting; (4) stooping, kneeling, or crouching; (5) reaching or extending your arms above shoulder level; (6) lifting or carrying weights over 5 kg, like a heavy bag of groceries; (7) picking up a small coin from a table? Then, we select another six specific indicators^2^ using the same methods to construct an instrumental activities of daily living (IADLs) index (Cronbach's α = 0.8149, overall KMO = 0.8444) ranging from 0 to 100, also with a higher value representing a better health condition. The details of factor loadings are presented in the Supplementary Material.

**Figure 1:**
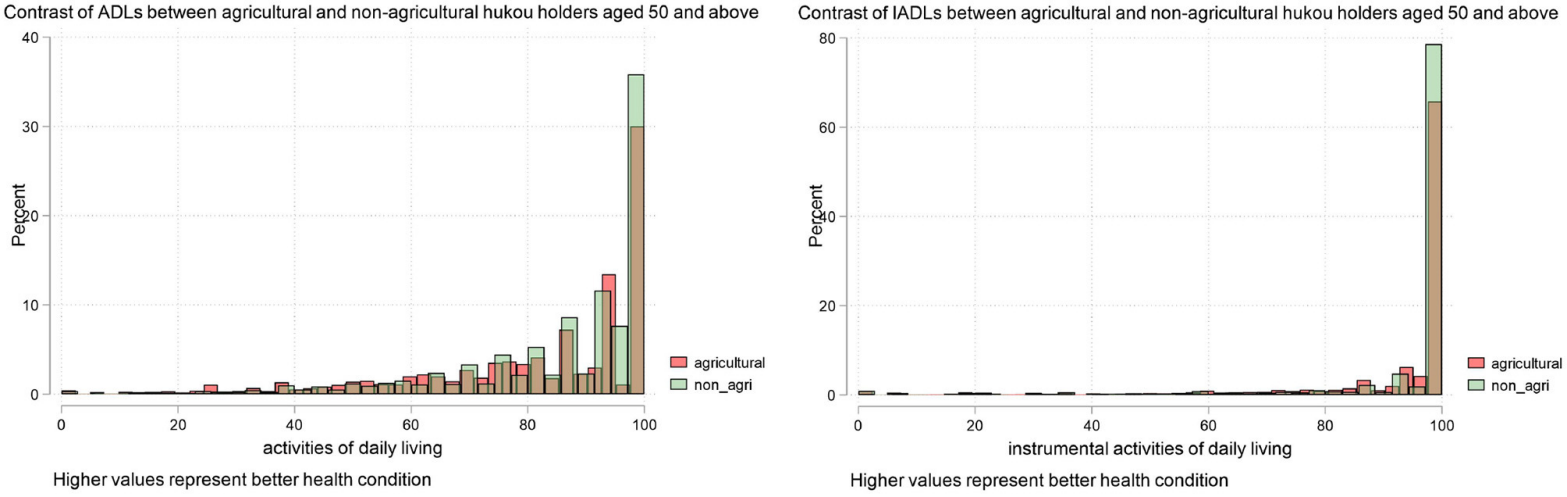
Contrast of health status between non-agricultural and agricultural hukou holders (left: ADLs index; right: IADLs index; samples have been restricted to those who are Han majority, and aged 50 and above).

Before we further employed an empirical technique, we first illustrate our dependent variables in Figure 1, it is obvious that non-agricultural hukou holders' health status is better than agricultural hukou holders.

## Empirical Strategy

### Benchmark Regression

As previously discussed, Chinese authorities imposed a strict birth-planning policy, i.e., the one-child policy on the Han majority in 1979, and many studies make rational and effective use of individuals' birth cohorts (i.e., born after 1979 or not) to explore the social influence of changing fertility (2, 21). We also utilize the birth year of individuals' first child to construct our instrumental variables (IVs), and draw support from two-stage least square (2SLS) to further investigate the health effect of lower fertility. The effectiveness of the IV method lies in the exogeneity of excluded IVs, anecdotal evidence suggests that the requirement is very likely to be satisfied in this case. As aforementioned, one-child policy enactment came as a shock to Chinese households. During the Cultural Revolution, they could not legitimately anticipate the sudden changing of fertility rights, and even though few officials had speculated the looming radical birth-planning policy, pregnancy lasts ~10 months, meaning that they were barely capable of having another child in such a pressing situation. Besides, cesarean section and other artificial birth control techniques were underdeveloped in the 1970s in mainland China. As a result, they were practically unable to manipulate the child birth cohort to avoid the unexpected strict one-child policy.

At the preliminary stage, the extent of rigidity and the specific launch time varied across mainland China, therefore, the discontinuity at the cutoff might be relatively small. As China's authority gradually promoted a series of decrees strengthening birth-planning, the one-child policy unfolded throughout mainland China^3^. So the change of fertility may not be fully reflected in the level (i.e., the intercept), and be expressed more adequately in the tendency (i.e., the slope). In other words, the level (jump) and slope (kink) would both change at the cutoff due to the different extent of exposure to the one-child policy. Generally, the later the birth year of people's first child, the higher probability of having only one child. Before 2011, having a second child was officially and nationally permitted for parents who did not have any siblings. Considering that individuals with a first child born before 1979 could conceive another child before the policy came into effect, to better identify the health effect of fertility, we use both jump and kink formed around the cutoff to construct our IVs: (1) *I*[*c*_*i*_ ≤ *c*_0_], which is an indicator function, if the first child of individual *i* had been born in and after 1979, it equals 1, before 1979, then it equals 0. (2) *I*[*c*_*i*_ ≤ *c*_0_] × (*c*_*i*_*-c*_0_), the interaction item combining *I*[*c*_*i*_ ≤ *c*_0_] with the difference of the birth year of the first child minus 1979.


(1) $$\begin{array}{l} only\text{\_}child_{i}=\beta_{1}I\left[c_{i}\geq c_{0}\right]+\alpha_{1}I\left[c_{i}\geq c_{0}\right]\times \left(c_{i}-c_{0}\right)\\ +\delta_{1}controls_{i}+\lambda_{p}+\varepsilon_{i} \end{array}$$

(2) $$\begin{array}{l} \left(i\right)adl\text{\_}index_{i}=\beta_{2}I\left[c_{i}\geq c_{0}\right]+\alpha_{2}I\left[c_{i}\geq c_{0}\right]\times \left(c_{i}-c_{0}\right)\\ +\delta_{2}controls_{i}+\lambda_{p}+\mu_{i} \end{array}$$

The two-stage least square (2SLS) technique has been used to identify the health effect of lower fertility. Equation (1) depicts the first stage estimation, *only_child*_*i*_ is the critical independent variable–whether people have only one child or not, if individual *i* only has one child, let *only_child*_*i*_ = 1, if he or she has multiple children, let *only_child*_*i*_ = 0. Equation (2) denotes the reduced-form specification we used earlier to construct the ADLs index and IADLs index as our dependent variables, respectively, to represent individuals' health status. We select 10 control variables presented as “*controls*_*i*_” in equations (1, 2): gender, age, marital status, and number of siblings as demographic variables; CPC membership, the degree of education, and health care insurance possession as socioeconomic variables; whether they smoke and the frequency of drinking alcohol as health behavior variables.

Local governments were given certain discretionary powers to adjust the rigidity of the enactment of the one-child policy based on local demographic and socioeconomic condition, which may results in different strictness of birth planning, and loosened grip on certain parents, we thus incorporate a provincial dummy variable in our estimation framework to capture the potential heterogeneity among different provinces, “λ_*p*_” is the provincial fixed effect. “μ_*i*_” and “ε_*i*_” represent the error term. The flexibility of imposing fertility controlling of local government may also loosen the ties restricting childbirth, so the proportion of parents with only one child is relatively lower, only 15%, which can be seen in Table 1. The 2SLS approach therefore provides an identification of health effect of having only one child instead of multiple children, which could be calculated as $\frac{\omega_{1}{\hat{\beta }}_{1}+\omega_{2}{\hat{\alpha }}_{1}}{\omega_{1}{\hat{\beta }}_{2}+\omega_{2}{\hat{\alpha }}_{2}}$, ω_1_ = *cov*(*D*_*i*_*, I*[*c*_*i*_ ≤ *c*_0_]), and ω_2_ = *cov*(*D*_*i*_*, I*[*c*_*i*_ ≤ *c*_0_] × (*c*_*i*_*-c*_0_)), which represent the weights reflecting the relative strength of the two IVs.

**Figure 2:**
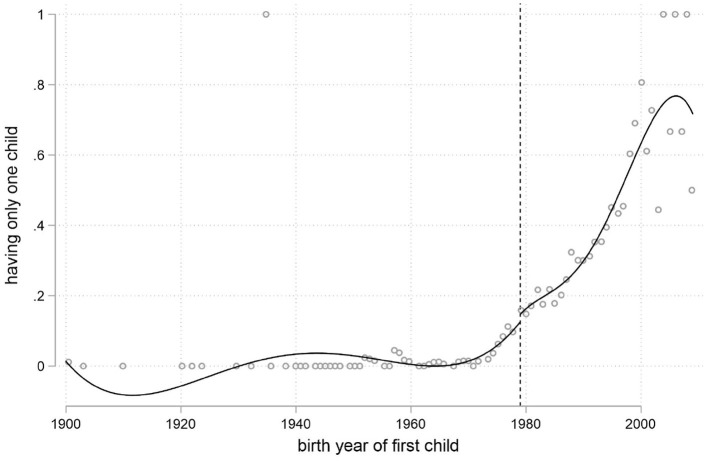
The impact of the one-child policy on individuals' fertility.

As illustrated in Figure 2, the intercept and slope of having only one child instead of multiple children for the Han majority both demonstrate an obvious change at 1979, especially for the change of slope, justifying our IVs construction.

Table 2 presents the results of the benchmark regression. The coefficients of our IVs in the even-numbered columns are all positive and significant at the 1% level, empirically justifying the strong correlation between having only one child and the jump and kink, and the minimum eigenvalues are all far greater than the critical value 19.93, rejecting the null hypothesis of the existing weak IV. Moreover, the magnitude of the coefficients of IVs in the urban group is noticeably higher than that in the rural group, this may be because families in several rural areas were permitted to have two children if the first one was a daughter in 1984 for a temporary period, besides, disobeying the one-child policy might cause more harm for urban citizens, since their career could be destroyed once they get caught having multiple children, especially when urban citizens have been working in public sectors. In columns (1) and (5), the coefficients of having only one child are both statistically insignificant, suggesting that lower fertility does not harm Chinese urban parents' health. By contrast, in columns (3) and (7), the coefficients of having only child for rural hukou holders are also both significantly negative, and the magnitude increases dramatically compared to the urban group, assuming that the imposition of the one-child policy dampened China's rural parents' health more.

**Table 2:** The health effect of having only one child.

**Variables**	**(1)**	**(2)**	**(3)**	**(4)**	**(5)**	**(6)**	**(7)**	**(8)**
	**urban_adl**		**rural_adl**		**urban_iadl**		**rural_iadl**	
	**adl_index**	**only_child**	**adl_index**	**only_child**	**iadl_index**	**only_child**	**iadl_index**	**only_child**
only_child	−1.085		−9.165^*^		−3.356		−12.083^***^	
	(2.743)		(3.827)		(2.355)		(3.256)	
I[c_i_ ≤ c_0_]		0.284^***^		0.061^***^		0.284^***^		0.060^***^
		(0.031)		(0.012)		(0.030)		(0.012)
I[c_i_ ≤ c_0_] × (c_i_-c_0_)		0.028^***^		0.019^***^		0.028^***^		0.019^***^
		(0.003)		(0.001)		(0.003)		(0.001)
Controls	Yes	Yes	Yes	Yes	Yes	Yes	Yes	Yes
Minimum eigenvalue	93.921		145.802		93.921		147.557	
Observations	1,677	1,677	6,149	6,149	1,677	1,677	6,154	6,154

*Standard errors in parentheses*;

*** *p < 0.001*,

* *p < 0.05*.

*For simplicity, the sample group of urban hukou holders is labeled as “urban,” and the sample group of rural hukou holders is labeled as “rural.” The samples are restricted to those aged between 50 and 80, have at least one child, and belong to the Han majority*.

### Mechanism Analysis

Next, we further investigate through which channel Chinese rural hukou holders bear the brunt of the lower fertility brought by the one-child policy. Scholars have emphasized the important role intergenerational transfers played in supporting elders in China (38, 39), and the amount of intergenerational transfers often correlated negatively with the number of children (26, 40). As shown in Table 3, we first examine the impact of having only one child on upstream intergenerational transfer, in columns (1) and (2), the dependent variable is the natural logarithm of the aggregate of the amount of financial support received from children during the last year, which consists of both pecuniary and in-kind payment-received support, and its coefficients are all significantly positive. The same 2SLS framework using both jump and kink as IVs is applied again, the coefficients of having only one child are negative and significant at the 1% level for both urban and rural hukou holders, but the magnitude of having only one child of the urban group only accounts for 38.5% of the rural group, suggesting that lower fertility depresses rural hukou holders' upstream intergenerational transfers more, therefore the health condition of rural hukou holders are more negatively responsive to lower fertility than their urban counterparts. Song and Smith (12) pointed out that rural hukou holders are always entitled to education resources inferior to their non-agricultural counterparts in their earlier life stage, and they often face occupational segregation and wage arrears in their adulthood. Consequently, for agricultural elders, the reduction in the number of children may metamorphose into lower upstream transfers than their non-agricultural peers.

**Table 3:** The mechanism analysis.

**Variables**	**(1)**	**(2)**	**(3)**	**(4)**	**(5)**	**(6)**	**(7)**	**(8)**
	**Urban**	**Rural**	**Urban**	**Rural**	**Urban**	**Rural**	**Urban**	**Rural**
	**lntrans_from**	**lntrans_from**	**housing**	**housing**	**mean_edu**	**mean_edu**	**mean_vispermon**	**mean_vispermon**
only_child	−3.605^***^	−9.388^***^	0.279^*^	0.003	1.774^**^	0.746	−9.111^***^	−6.909^**^
	(0.784)	(0.973)	(0.123)	(0.150)	(0.559)	(0.594)	(2.228)	(2.118)
Controls	Yes	Yes	Yes	Yes	Yes	Yes	Yes	Yes
Observations	1,428	5,716	1,670	6,141	1,543	5,991	1,421	5,685

*Standard errors in parentheses*;

*** *p < 0.001*,

** *p < 0.01*,

* *p < 0.05*.

*For simplicity, the sample group of urban hukou holders is labeled as “urban,” and the sample group of rural hukou holders is labeled as “rural.” The samples are restricted to those aged between 50 and 80, have at least one child, and belong to the Han majority*.

While, having fewer children could spare more resources redirected to parents to improve their life quality, next, we explore how lower fertility affects parental housing condition. In columns (3) and (4), the dependent variable is housing condition factor^4^ rotated through the same factor analysis method previously conducted to construct ADLs and IADLs indexes, with a higher value indicating a better housing condition. The coefficient of having only one child is positive and significant at the 5% level for the urban group, suggesting that raising fewer children did translate into better housing condition for urban parents, which therefore might curb the negative health effect of lower fertility, while, rural elders may be less lucky, the coefficient of only having one child is statistically insignificant for rural parents, and its magnitude plummets drastically compared with their urban counterparts. Given the consistently inferior socioeconomic status linked with rural hukou, it is unsurprising that elders are unable to improve their housing condition in rural areas despite the decline in number of children they have to raise.

As aforementioned, the “quantity-quality tradeoff” theory may play a role in improving offspring's educational attainment within a smaller family, thus providing a chance for elderly parents to benefit from their children's higher educational achievement (24). In columns (5) and (6), we use children's average number of years of education as the dependent variable, the coefficient of having only one child in 2SLS estimation is positive and significant at the 1% level in the urban group, in contrast, its magnitude and statistical significance both plunge for rural elderly parents, implying that diminishing fertility may not effectively transform into increased educational attainment in China's rural areas. Hu (14) finds that receiving elementary education could effectively improve China's urban citizens' health condition, by comparison, the positive health effect of education becomes statistically significant for rural residents only when they finished senior high school. The relatively inferior education quality and infrastructure concomitant with rural hukou and cumulative dis/advantage theory may lead to impairment of the efficiency of the “quantity-quality tradeoff” theory in China's rural families, and undermine the potential advantage of having fewer children for rural elders.

The deterrent effect of lower fertility on the contacts and visits from children are also well-documented by economists (24, 33). We next choose the average number of days receiving visits from children per month as our dependent variable. In columns (7) and (8), the coefficients of having only one child rather than multiple children are both statistically negative, indicating that a downsized number of children trims the frequency of vis-à-vis contact between parents and offspring. The magnitude and statistical significance are both more pronounced for the urban group, considering the hard-pressed lifestyle adopted in many of China's cities, it is natural that adult children would put more energy and time in the labor market, thus reducing the amount of time visiting parents.

Through exploring various potential mechanisms, Table 3 presents a relatively mixed result of how having fewer children affects elderly parents, on the one hand, dwindled childbirth paired with upstream intergenerational transfer, dragged down the frequency of visits from offspring for both urban and rural parents; on the other hand, it increased children's educational attainment and improved parental housing condition, while, the positive effects are only statistically significant for the urban group in the 2SLS framework, which may shed light on why raising fewer children only dampened rural parents' health.

### Heterogeneous Analysis

In this section, we construct interactive items to analyze the heterogeneity of having only one child on peoples' health. The extended regression model (ERM) has been used in this part. ERM could estimate the interactions of endogenous covariates, and interactions of endogenous with exogenous covariates (41), which earlier STATA commands like ivregress, ivprobit, and ivtobit could not. In addition, Jiang and Wang (42) point out that bidirectional causality, omitting variables, and sample selection bias could be addressed simultaneously in ERM. Since our previous results demonstrate that the health effect of having only one child instead of multiple children only exhibits statistical significance for rural elderly parents, we principally analyze the potential heterogeneity within the rural group in this subsection. As Table 4 shows, rural female elders tend to benefit more from lower fertility. Mothers often have a more intimate connection with children, the relatively higher emotional support may dilute the negative health effect of lower fertility at some point, moreover, the motherhood penalty in the labor market has been well-documented by scholars (30, 31), raising fewer children might boost mothers' labor market performance and increase their income, therefore acting as a cushion against the potential negative impact of having only one child in the old-age stage. In addition, as the direct bearer of pregnancy, mothers often bear the brunt of the negative health effects of giving birth, diminished fertility would thus naturally lower the risks facing women (43, 44). Meanwhile, the coefficients of the interaction term are all statistically insignificant in columns (2–8), suggesting universality of our earlier results.

**Table 4:** The heterogeneous analysis.

**Variables**	**(1)**	**(2)**	**(3)**	**(4)**	**(5)**	**(6)**	**(7)**	**(8)**
	**ERM**							
	**adl_index**	**iadl_index**	**adl_index**	**iadl_index**	**adl_index**	**iadl_index**	**adl_index**	**iadl_index**
only_child × female	2.687+	1.126						
	(1.503)	(1.268)						
only_child × age			−0.003	0.118				
			(0.118)	(0.100)				
only_child × edu_level					−1.148	0.260		
					(0.896)	(0.756)		
only_child × single							2.359	1.318
							(2.004)	(1.694)
Controls	Yes	Yes	Yes	Yes	Yes	Yes	Yes	Yes
Observations	6,149	6,154	6,149	6,154	6,149	6,154	6,149	6,154

*Standard errors in parentheses*;

+ *p < 0.1*.

*For simplicity, the sample group of urban hukou holders is labeled as “urban,” and the sample group of rural hukou holders is labeled as “rural.” The samples are restricted to those aged between 50 and 80, have at least one child, and belong to the Han majority*.

### Further Discussion

China has seen its hukou system experience a variety of reforms, which gradually allowed rural residents to migrate to cities as reform and opening-up unfolded throughout mainland China and the market regained its ascendancy of signaling permission. While, the accessibility of multifarious socioeconomic resources determined by the hukou category may not vary very much, rural residents without local urban hukou could still be denied schooling, housing, and medical care after they migrate to cities, which retains the urban-rural divide bestriding China's society (12, 17). In 2014, CHARLS provided Life History data, we draw people's initial hukou status from this data set and identify those who have experienced rural-to-urban hukou conversion, to further explore the health effect of having fewer children more thoroughly. As Table 5 presents, in columns (1) and (2), only having one child barely affects parental health for those who were born with urban hukou. In comparison, the coefficients of having only one child rather than multiple children are both significantly negative in columns (3) and (4), revealing the deterrent impact of decreased fertility on the health condition for people whose first hukou belongs to the rural category. What is more intriguing is the results in columns (5) and (6), for those who initially had rural hukou but later changed their hukou to the urban category, even though the magnitude of the coefficients of having only one child exhibits an uptick compared with columns (1) and (2), the statistical significance still remains absent, assuming that overcoming the hukou barrier would effectively dilute the negative impact of lower fertility. Considering that Confucian culture has long been ingrained in China, and rural areas especially emphasize obedience and filial piety, having fewer children might be seen as defiance to family and clan. Silverstein et al. (45) notice that conforming to prevailing cultural norms has a health-enhancing effect, therefore having fewer children may pose more of a burden on rural residents' health. Once the hukou threshold has been surmounted, several factors including waning pressure from family, urbanization overtaking traditional culture, and improved resources concomitant with urban hukou combined to result in counteracting the negative influence from lower fertility.

**Table 5:** The health effect considering initial hukou and rural-to-urban conversion.

**Variables**	**(1)**	**(2)**	**(3)**	**(4)**	**(5)**	**(6)**
	**initial_urban**		**initial_rural**		**rural-to-urban conversion**	
	**adl_index**	**iadl_index**	**adl_index**	**iadl_index**	**adl_index**	**iadl_index**
only_child	0.461	−1.757	−7.320^*^	−9.502^***^	−2.941	−3.610
	(3.521)	(2.658)	(3.211)	(2.748)	(4.507)	(3.963)
Controls	Yes	Yes	Yes	Yes	Yes	Yes
Observations	584	584	6,569	6,572	843	843
R-squared	0.196	0.190	0.191	0.073	0.223	0.095

*Standard errors in parentheses*;

*** *p < 0.001*,

* *p < 0.05*.

*For simplicity, the sample group of those whose initial hukou is urban-labeled is marked as “initial_urban,” the sample group of those whose initial hukou is rural-labeled is marked as “initial_rural,” and the sample group of those who have changed from rural hukou to urban hukou is labeled as “rural-to-urban conversion.” The samples are restricted to those aged between 50 and 80, have at least one child, and belong to the Han majority*.

### Robustness Check

In this subsection, we provide several robustness checks. Considering that previously used dependent variables are obtained through factor analysis, we firstly substitute the construction method with directly summing specific indicators to examine the health effect of only having one child rather than multiple children on parental health. As aforementioned, ADLs consist of seven indicators, and IADLs consist of six, so the range of the sum of ADLs and IADLs is 28 and 24, respectively, and with a higher value indicating a better health status.

As Table 6 shows, the estimation results of first-stage estimation are identical with the benchmark regression in Table 2, and the effect of decreased fertility on parental health demonstrates the same pattern, the coefficients of only having one child are both negative and significant in columns (3) and (7), respectively, revealing the deterrent effect of having fewer children on China's rural elders. In comparison, the coefficients of having only one child are both statistically insignificant in columns (1) and (5), showing again that lower fertility barely affects urban parents' health, which aligns with our benchmark regression, and suggests that the results are insensitive to the method of constructing the dependent variables adopted.

**Table 6:** The robustness check (1).

**Variables**	**(1)**	**(2)**	**(3)**	**(4)**	**(5)**	**(6)**	**(7)**	**(8)**
	**urban_adl**		**rural_adl**		**urban_iadl**		**rural_iadl**	
	**adl_index**	**only_child**	**adl_index**	**only_child**	**iadl_index**	**only_child**	**iadl_index**	**only_child**
only_child	−0.211		−1.863^*^		−0.555		−2.456^***^	
	(0.600)		(0.822)		(0.411)		(0.595)	
I[c_i_ ≤ c_0_]		0.284^***^		0.061^***^		0.284^***^		0.060^***^
		(0.031)		(0.012)		(0.030)		(0.012)
I[c_i_ ≤ c_0_] × (c_i_-c_0_)		0.028^***^		0.019^***^		0.028^***^		0.019^***^
		(0.003)		(0.001)		(0.003)		(0.001)
Controls	Yes	Yes	Yes	Yes	Yes	Yes	Yes	Yes
Minimum eigenvalue	93.921	145.802	93.921	147.557
Observations	1,677	1,677	6,149	6,149	1,677	1,677	6,154	6,154

*Standard errors in parentheses*;

*** *p < 0.001*,

* *p < 0.05*.

*For simplicity, the sample group of urban hukou holders is labeled as “urban,” and the sample group of rural hukou holders is labeled as “rural.” The samples are restricted to those aged between 50 and 80, have at least one child, and belong to the Han majority*.

Commonly used local average treatment effect (LATE) estimated by IVs methods only solve selection bias on levels, however, the problems of selection on both levels and gains often occur in more reasonable cases (46). In this article, this means that more motivated people tend to decide on having only one child instead of multiple children as the potential gains could be expected, this selection on returns is called essential heterogeneity, so we next employ marginal treatment effects (MTEs) to further explore the health effect of having only one child on non-agricultural and agricultural hukou holders. Andresen (46) points out that MTEs could capture the unobserved resistance to treatment (in this case, having only one child), the expectation of higher gains from being treated underpins the lower resistance to treatment, thus catalyzing certain people with lower resistance into the treatment group.

MTEs are based on the generalized Roy model:


(3) $$\begin{array}{c} Y_{j}=\mu_{j}\left(X\right)+U_{j},\;j=1,2 \end{array}$$

(4) $$\begin{array}{c} Y=DY_{1}+\left(1-D\right)Y_{0} \end{array}$$

(5) $$\begin{array}{c} D=1\left\{\mu_{D}(Z)>U_{D}\right\},\;Z=\left(X,Z\_\right) \end{array}$$

Where *Y*_1_ and *Y*_0_ are the potential outcomes in the treated and untreated state, respectively. They are both modeled as functions of observed explanatory variables *X*. Equation (5) is an indicator function which is a reduced-form way of modeling selection into treatment as a function of observed *X* in equation (1) and excluded IVs *Z*_. *U*_*D*_ represents the quantile of unobserved resistance to treatment, which can be normalized to a uniform distribution on the unit interval. Thus, μ_*D*_(*Z*) can be interpreted as the propensity score measuring the conditional probability of entering the treatment.

There are usually two ways to estimate MTEs: (1) a local IV, which recognizes MTE as the derivative of the conditional expectation of *Y* regarding the propensity score; and (2) a separate approach, which estimates the conditional expectations of *Y*_1_ and *Y*_0_ in the treated and untreated samples separately (in this case, *Y*_1_ and *Y*_0_ represent the health status of parents with only one child and multiple children, respectively). Brinch et al. (47) point out that local IV cannot identify the linear MTE model with a binary instrument, so we only selected the “jump” as our IV in this part, and use the separate approach to perform the MTE estimation.


(6) $$\begin{array}{c} E\left(Y_{1}|X=x,D=1\right)=x\beta_{1}+E\left(U_{1}|U_{D}\leq p\right)=x\beta_{1}+K_{1}\left(p\right) \end{array}$$

(7) $$\begin{array}{c} E\left(Y_{0}|X=x,D=0\right)=x\beta_{0}+E\left(U_{0}|U_{D}\leq p\right)=x\beta_{0}+K_{0}\left(p\right) \end{array}$$

(8) $$\begin{array}{c} \begin{array}{c} MTE\left(x,u\right)=E\left(Y_{1}|X=x,U_{D}=u\right)-E\left(Y_{0}|X=x,U_{D}=u\right)\\ MTE\left(x,u\right)=x\left(\beta_{1}-\beta_{0}\right)+k_{1}\left(u\right)-k_{0}\left(u\right) \end{array} \end{array}$$

In equations (6, 7), *D* denotes the treatment status (having only one child = 1, having multiple children = 0), *K*_*j*_(*p*) is the control function capturing the essential heterogeneity, the separate approach could control selection through *K*_*j*_(*p*), which is in line with Heckman selection (46), and *k*_*j*_(*u*) = E(*U*_*j*_ | *U*_*D*_ = *u*). We demonstrate commonly used treatment effect parameters provided by MTEs in Table 7, ATT does not exhibit much variation between the urban and rural groups, LATE in the rural group is significantly negative, which is consistent with the previous 2SLS estimation; lower fertility tends to put more pressure on rural hukou holders' health condition.

**Table 7:** The robustness check (2).

**Variable**	**(1)**	**(2)**	**(3)**	**(4)**
	**Urban**	**Rural**	**Urban**	**Rural**
	**adl_index**	**adl_index**	**iadl_index**	**iadl_index**
Average treatment effect (ATE)	2.442	−10.071	1.059	−8.889
	(3.438)	(7.696)	(2.895)	(6.513)
Average treatment effect on the treated (ATT)	−6.806+	−11.514^**^	−9.841^**^	−9.706^**^
	(3.548)	(4.021)	(2.989)	(3.406)
Average treatment effect on the untreated (ATU)	7.350	−9.912	6.846+	−8.802
	(4.794)	(8.542)	(4.038)	(7.231)
Local average treatment effect (LATE)	−1.881	−12.231^**^	−3.043	−9.396^**^
	(2.999)	(4.271)	(2.526)	(3.631)
Controls	Yes	Yes	Yes	Yes
Observations	1,665	6,090	1,665	6,095

*Standard errors in parentheses*;

** *p < 0.01*,

+ *p < 0.1*.

*For simplicity, the sample group of urban hukou holders is labeled as “urban,” and the sample group of rural hukou holders is labeled as “rural.” The samples are restricted to those aged between 50 and 80, have at least one child, and belong to the Han majority*.

**Figure 3:**
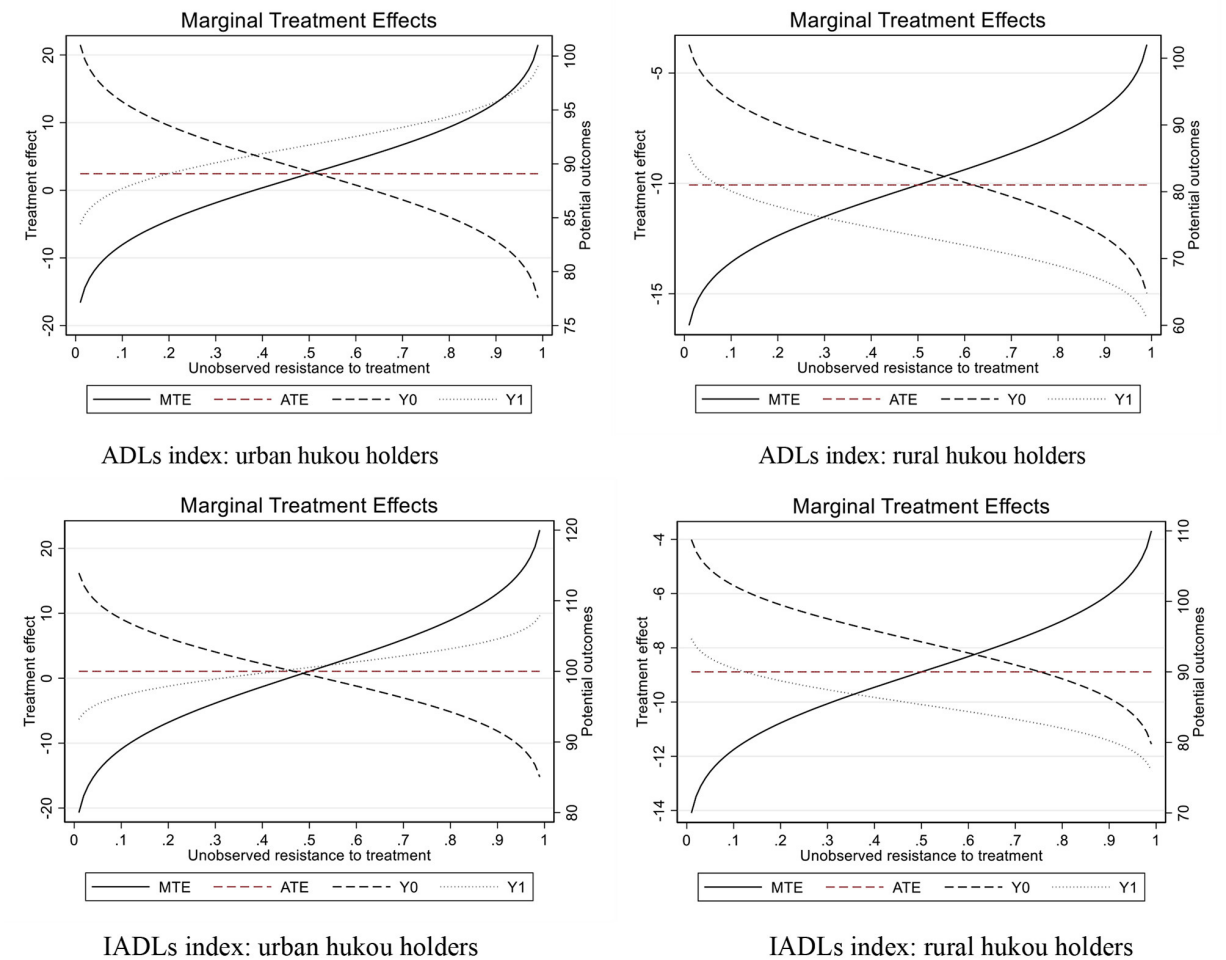
The resulting potential outcomes from MTE.

We also illustrate the MTEs and relevant results in Figure 3. ATEs of the rural group are clearly below the urban group in both cases of explained variables being the ADLs and IADLs indexes, implying that having only one child instead of multiple children may dampen rural hukou holders' health more. The upward sloping pattern of MTE suggests that people with lower resistance bear more of the health burden of lower fertility. Furthermore, the resulting potential outcomes delineate individuals' health condition alongside the resistance to treatment. The MTE is the difference between *Y*_1_ and *Y*_0_, so we can examine whether the upward sloping pattern of MTE plotted in Figure 3 is created by upward sloping *Y*_1_ or downward sloping *Y*_0_, or a combination. As illustrated in Figure 3, in the urban group, there is a combination of upward *Y*_1_ and downward *Y*_0_, suggesting that urban citizens with only one child and higher resistance have a better health condition, and those who have multiple children and lower resistance have better health. In comparison, the rural group demonstrates both downward potential outcomes, assuming rural residents with higher resistance have worse health.

For consistency, in the last part, we also only use “jump”–individuals' first child is born in or after 1979 as our IV, and substitute having only child or multiple children with the number of sons and daughters as our core explanatory variables to construct 2SLS estimation. As shown in Table 8, the coefficients of the number of sons and daughters are all positive in the rural group, showing that having multiple children instead of only one child could improve rural parents' health condition. Moreover, no matter which explained variables we choose (ADLs or IADLs index), no matter what the gender of individuals' children (son or daughter), the coefficients of the number of offspring in the rural group always demonstrate higher magnitude and statistical significance compared to the urban group, suggesting that relatively higher fertility may benefit Chinese rural hukou holders more, this agrees with previous research; rural hukou holders are mainly biased toward choosing children as the basis for future support in their old age (16).

**Table 8:** The robustness check (3).

**Variables**	**(1)**	**(2)**	**(3)**	**(4)**	**(5)**	**(6)**	**(7)**	**(8)**
	**Urban**	**Urban**	**Rural**	**Rural**	**Urban**	**Urban**	**Rural**	**Rural**
	**adl_index**	**adl_index**	**adl_index**	**adl_index**	**iadl_index**	**iadl_index**	**iadl_index**	**iadl_index**
Son	0.157		7.076^**^		0.048		4.895^*^	
	(2.737)		(2.554)		(2.340)		(4.718)	
Daughter		0.195		11.532^*^		0.059		8.004^*^
		(3.385)		(4.869)		(2.895)		(3.867)
Observations	1,677	1,677	6,150	6,708	1,677	1,677	6,155	6,155

*Standard errors in parentheses*;

** *p < 0.01*,

* *p < 0.05. For simplicity, the sample group of non-agricultural hukou holders is labeled as “non-agriculture,” and the sample group of agricultural hukou holders is labeled as “agriculture.” The samples are restricted to those aged between 50 and 80, have at least one child, and belong to the Han majority*.

## Conclusion

Since China's authority promoted the one-child policy in 1979, the number of one-child families has continued to rise. In recent years, as parents with only one child enter into their old-age stage, how does their health compare with parents having multiple children? Combining the background of urban-rural duality, this paper uses the one-child policy as an exogenous shock to construct IVs, and examines the health effect of having only one child rather than multiple children for urban and rural hukou holders aged between 50 and 80. The empirical results show that: (1) Having only one child instead of multiple children significantly depresses rural parents' health, while urban parents are relatively immune to this negative health impact. (2) Our mechanism analysis suggests that only having one child scaled down the upstream intergenerational transfer payments, and the received financial support of rural parents creates more pressure; the “quantity-quality tradeoff” theory is chiefly efficient in promoting the educational attainment for urban only children; similarly, having fewer children principally improves urban parents' housing condition; while, lower fertility trims the frequency of visits from offspring both for urban and rural parents, and urban parents tend to receive fewer visits than their rural counterparts with only one child. Therefore, raising fewer children may dampen rural parents' health more. (3) The heterogeneous analysis finds that the negative health effect of having only one child is relatively moderate for rural female residents, we attribute this heterogeneity to more intimacy between mothers and children, motherhood penalty in the labor market, and the risk ensued from pregnancy and giving birth.

This article carries important implications. The hukou system colonizes its role of allocating resources across mainland China, which has disadvantaged rural hukou holders for a long time. Rural hukou holders are always at a disadvantage in accessing various resources, such as housing, medical care, education, etc. Consequently, their health status is worse than urban hukou holders. Many scholars find that rural parents tend to rely on their children providing old-age support in their late life stage (16, 48). In recent years, as the aging process has continued to accelerate throughout China, as more and more one-child parents are approaching old age, and as rural parents with only one child face the dual scarcity of socioeconomic resources and upstream intergenerational support, their health condition deserves more attention from the government. In addition, China's countryside zones are often imbued with emphasis on conformity and filial piety, which definitely exacerbates the negative health effect of decreased fertility, in contrast, urbanization could slash the cost of disobeying cultural norms, and urban elders often have larger pensions thus rely less on their offspring to provide support in old age than their rural counterparts. On the one hand, China's authority should further ease or even neutralize the connection between individuals' hukou category and the accessibility of socioeconomic resources as well as facilitate urbanization; on the other hand, government should enhance old-age support for parents with only one child, especially for rural parents, their compliance to the one-child policy deserves more social care and public welfare, which would serve as a cushion against the negative health effect of lower fertility.

## Data Availability Statement

Publicly available datasets were analyzed in this study. This data can be found at: The data of CHARLS 2018 is publicly available by application through http://charls.pku.edu.cn.

## Author Contributions

CL and JH: conceptualization. JH: methodology. JH, CL, and CY: writing-original draft preparation. CL and CY: writing-review and editing. All authors contributed to the article and approved the submitted version.

## Funding

This research was funded by the Shanghai Social Science Planning Project, Grant Number 2019BJL004.

## Conflict of Interest

The authors declare that the research was conducted in the absence of any commercial or financial relationships that could be construed as a potential conflict of interest.

## Publisher's Note

All claims expressed in this article are solely those of the authors and do not necessarily represent those of their affiliated organizations, or those of the publisher, the editors and the reviewers. Any product that may be evaluated in this article, or claim that may be made by its manufacturer, is not guaranteed or endorsed by the publisher.

## Footnotes

^1^Proletariat Cultural Revolution, launched by Chairman Mao Zedong in 1966, aimed to maintain the purity of the Party and seek China's own road to building socialism initially, leading to a decade long violence, social turmoil, and stagnation of economic development throughout mainland China until 1976.

^2^Because of health and memory problems, do you have any difficulties with (1) doing household chores; (2) preparing hot meals; (3) shopping for groceries; (4) making phone calls; (5) taking medications; (6) managing your money, such as paying your bills, keeping track of expenses, or managing assets?

^3^For example, in September 1982, the 12th National Congress of the Communist Party of China (CPC) established family planning as a basic national policy, and it was written into the Constitution in December of that year. In May 1991, the Central Committee of the CPC and the State Council made the *Decision on Strengthening Family Planning and Strictly Controlling Population Growth*, clearly enacting the birth-planning policy and strictly controlling population growth. In September 2002, the *Population and Family Planning Law of the People's Republic of China* came into effect, etc. Although there existed a temporary period of time in which families in certain rural areas were allowed to have a second child if the first one was a daughter in 1984, the central government later felt the laxity and call for stronger compliance to birth-planning, then proclaimed the *Central Document 13* in 1986 more rigorously regulating the one-child policy than before.

^4^We selected seven variables (overall KMO value = 0.7626) to construct a housing condition factor: the type of structure of the current residence (concrete = 1, others = 0); the type of toilet (with a seat = 1, without a seat = 0); whether current residence has electricity (yes = 1, no = 0); whether current residence has running water (yes = 1, no = 0); whether current residence is equipped with an in-house shower or bath facility (yes = 1, no = 0); whether current residence has a coal gas or natural gas supply (yes = 1, no = 0); and whether current residence has a broadband Internet connection (yes=1, no=0). The factor analysis using the aforementioned iterated communalities were employed again to rotate the housing condition factor.
