# Supplementary material for: The Health Effect of the Number of Children on Chinese Elders: An Analysis Based on Hukou Category
Source: Front Public Health. 2021 Nov 16;9:700024. doi: 10.3389/fpubh.2021.700024 (PMC8634947; doi:10.3389/fpubh.2021.700024)
Supplement: Supplementary file 1 [file Table_1.docx]

**TABLE 1|** Rotated Factor Loadings.

| Variable | Factor_ADLs | Variable | Factor_IADLs |
| --- | --- | --- | --- |
| jogging | 0.5906 | chore | 0.8140 |
| chair | 0.6574 | meal | 0.8236 |
| stairs | 0.7317 | shopping | 0.7944 |
| stoop | 0.7165 | phone | 0.4835 |
| arms | 0.5458 | medication | 0.6040 |
| carry_weight | 0.6535 | manage_money | 0.6722 |
| pick_coin | 0.4176 |  |  |
